# *Xtal-xplore-R*: a graphical tool for exploring the residual function involved in crystal structure determination

**DOI:** 10.1107/S1600576715012169

**Published:** 2015-07-28

**Authors:** Jan Marten Simons, Georg Roth

**Affiliations:** a Institute of Crystallography, RWTH Aachen University, Germany

**Keywords:** residual functions, crystal structure determination

## Abstract

This work presents *Xtal-xplore-R*, a tool dedicated to the visualization of two-dimensional cuts through the multidimensional crystallographic residual function. It imports arbitrary crystal structures, generates artificial diffraction data, and calculates and investigates the residual function in parameter space. The program serves two major purposes. Firstly, it is part of a more general project dealing with structure determination *via* global optimization techniques. In this context, the tool is being used to systematically analyse characteristic universal features of the target function (residual function) which can be used to develop appropriate problem-specific heuristic optimization algorithms. Secondly, *Xtal-xplore-R* is intended as a didactic tool to visualize how changes in atom parameters affect the residual function and can be used to demonstrate manual structure optimization for simple crystal structures.

## Abstract 2

Using a newly created open-source tool *Xtal-xplore-R* some universal features of the crystallographic residual function have been found, which might help in developing new algorithms suitable for crystal structure solution.

## 1. Motivation   

### 1.1. Structure determination from incomplete data   

The development of methods for the determination of crystal structures from incomplete (single-crystal or powder) X-ray diffraction data (XRD) has been a major topic in structural research for many decades. Increased computational capabilities in hardware and new algorithms have led to increasing success in this field.

Classical methods of crystal structure determination like the Patterson method or direct methods require the knowledge of individual structure factor amplitudes. Complete sets of such data are not available in the powder case, even if high-resolution synchrotron data are being used. Yet, the determination of small-to-medium-sized crystal structures from such incomplete data has been quite successful during the past few years.

More recently, dual space density-modification techniques (charge flipping) have been introduced (for a review, see Palatinus, 2013 ▸). In particular, organic structures can be solved with approaches exploiting the approximately known structure of the molecule(s) and a combination of energy minimization and rigid-body refinement, as shown by Schmidt & Dinnebier (1999 ▸), for example. Inorganic structures, on the other hand, pose a far greater problem as the absence of *a priori* connectivity information renders the application of such methods impossible.

Crystal structures with few atoms in the asymmetric unit of the unit cell and/or high space-group symmetry can be solved with little reference to diffraction data at all by systematically assigning Wyckoff positions to matching unit-cell content (Deng & Dong, 2009 ▸, 2011 ▸).

Fischer and Kirfel have suggested another approach, using projection techniques and the symmetry of iso-surfaces to reduce the phase volume of the parameter space (Fischer *et al.*, 2005 ▸; Zimmermann & Fischer, 2009 ▸). Yet, their approach seems most suitable for high-resolution single-crystal (neutron or synchrotron) diffraction data.

### 1.2. Structure determination *via* global optimization   

*Xtal-xplore-R* is part of a larger effort to apply global optimization algorithms to the task of structure determination. In this context the task of determining a crystal structure from (inevitably incomplete^1^) diffraction data is treated as a (global) optimization problem. The input data for the optimization are experimental diffraction data in the form of structure factor amplitudes 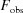 for each observation *hkl*. 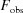 are essentially the square roots of the measured reflection intensities 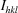 after suitable corrections. The second ingredient is a model equation that allows the calculation of corresponding quantities 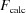. This is the classical structure factor equation, which is at the heart of the kinematical diffraction theory and is based on a Fourier transform of the electron density within the unit cell: 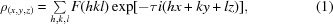


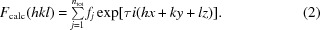



In these equations 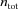 is the total number of scatterers in the unit cell, ρ is the electron density, 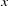, 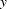, 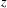 are fractional coordinates in direct space, 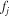 is the atomic form factor of scatterer *j*, 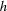, 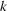, 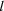 are the Miller indices of the observation *hkl*, and, for good reasons (Palais, 2001 ▸; Hartl, 2013 ▸), we use the notation of 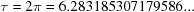.

From these two quantities (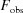 and 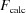), a target function (the crystallographic residual function) is calculated according to 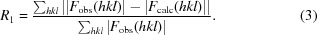



The task is then to optimize (minimize) this function with respect to the structural parameters that enter the structure factor equation. These are at least an overall scale factor that puts the observed data (which are on a relative scale) on the absolute scale of the calculated data, and the three-dimensional coordinates 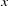, 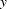, 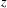 of the 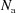 atoms in the asymmetric sector of the unit cell (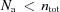 for all space groups except *P*1). Also, the atom type, represented by the atomic form factor 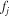, and an isotropic displacement parameter need to be assigned to each atom but are usually not optimized.

The dimensionality of the optimization problem is therefore of the order of 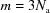. With typical values of 5–20 unique atoms for nontrivial crystal structures to be solved, the optimization problem is thus defined in a 15- to 60-dimensional parameter space. This type of optimization problem is probably one of the most frequently solved such multidimensional tasks in solid state research and is usually referred to as ‘structure refinement’. Local optimizers (usually least-squares algorithms) are used and, as such, the refinement requires a very good set of starting parameters for the optimization to converge to the true solution.

The global optimization task, in the absence of any suitable initial set of approximate coordinates, is much harder to solve: Assuming that a resolution of 0.1 along each parameter direction (coordinates normalized to the interval [0…1[) would be sufficient (see discussion of resolution below), a total of 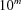 grid points would be needed to sample parameter space. This is not just a vast number;^2^ the problem size also increases exponentially with increasing number of atoms. For a detailed discussion on the complexity of the global optimization problem see Roth *et al.* (2011 ▸).

In a very general sense, the target function defined by equation (3) has the following characteristics:

(*A*) It is multidimensional (see above).

(*B*) It is multi-modal: the number of minima depends on the data resolution,

(*C*) It is band limited: the obvious reason is that equation (1) is defined *via* a Fourier transform with a finite number of accessible Fourier coefficients.

(*D*) The variables are not separable (a direct consequence of the underlying Fourier transform).

(*E*) It is noisy because the experimental intensities are subject to the counting statistics.

Most (if not all) global optimization algorithms will fail already because of characteristics (*A*) and (*B*). For instance, the ‘branch and bound’ type of algorithms [first applied to discrete optimization problems by Land & Doig (1960 ▸)] as well as ‘interval arithmetic’ approaches (Hansen, 1992 ▸; Kearfott, 1996 ▸) suffer from the huge number of branches/intervals that need to be evaluated before a decision can be made that a given parameter space volume cannot contain an extremum. In high dimensions these algorithms typically run out of storage before reaching definite decisions.

Random optimization procedures [random start plus local optimization, simulated annealing *etc*., see for instance Gelatt *et al.* (1983 ▸)], on the other hand, run out of time before they reach the global optimum with any suitable certainty.

Heuristic approaches are, therefore, unavoidable. For those to succeed, it is essential to explore and make use of possible *a priori* knowledge about characteristic features of the target function. Such characteristic features indeed exist, as we will show below, and these inspire effective optimization algorithms, which will be the subject of another publication.

## 2. *R*-factor landscapes   

To find such heuristics, we chose to take a look at the target function by generating ‘*R*-factor landscapes’:

An unknown crystal structure can be referred to as determined from its diffraction pattern when the position and type of all of its scatterers have been found. In this case a simulated diffraction pattern of the structure should match the diffraction pattern (perfectly).

To quantify the goodness of a match, the crystallographic residual function *R* (*R* factor) is used as a measure [see equation (3)]. An *R* factor of zero denotes a perfect match of structure factors, while two random distributions of the same scatterers usually give an *R* value of around ∼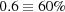 (McMahon, 2008 ▸). 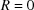 can only be reached when refining against simulated structure factors or intensities, while in practice a good refinement of a crystal structure against X-ray single-crystal diffraction data from a laboratory experiment usually can reach *R* values of a few percent, and X-ray powder diffraction data usually only allow for 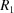 between 5 and 15% for a good Rietveld refinement (Rietveld, 1969 ▸).

Visualization of the multidimensional residual function 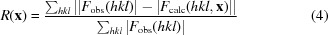
[with 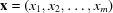 being the multidimensional fractional coordinate vector of all scatterers] is extremely hard.

Yet, a two-dimensional section through the *m*-dimensional parameter space is still useful for visualization purposes, provided the parameters chosen for the cut are representative for all the other ones. This, indeed, applies to the atom coordinates, which all enter the structure factor formula (2) in a similar way. The contribution of individual atom *j* to the sum in equation (2), however, is weighted by the form factor 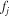, and this influence on the target function will be discussed further below.

## 3. *Xtal-xplore-R*   

To calculate the two-dimensional cuts through the aforementioned parameter space we created a graphical tool called *Xtal-xplore-R*.

*Xtal-xplore-R* uses CIF as a standard file format to load crystal structure data (*via iotbxcif*; Gildea *et al.*, 2011 ▸). Right now *Xtal-xplore-R* operates only on simulated single-crystal data, but future extensions will enable it to also work with powder data or user-supplied intensity data of any sort. To generate the plots of the two-dimensional sections the program keeps track of two crystal structures. The first of these is called the ‘trial structure’, while the second one is called the ‘target structure’. As both structures are automatically transformed into space group *P*1 on loading, their atomic coordinates can be freely modified by the user.

A single two-dimensional cut is obtained by evaluating the target function (4) on a two-dimensional grid with 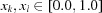 and increments of 0.01 (or 0.1) in each parameter direction while leaving all other parameter values 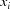 constant.

For the present purpose (visualization and study of the crystallographic residual function), the 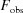 in equation (4) are replaced by calculated reference values for the target structure. So 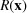 becomes 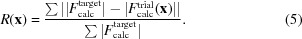
To obtain these 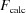 data the structure factor calculation module of the *Computational Crystallographic Toolbox* (*cctbx*) (Grosse-Kunstleve *et al.*, 2002 ▸, 2014 ▸) is used to generate the appropriate values using direct summation of partial structure factors that in turn are calculated from the atomic form factors, 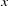, 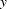, 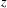 coordinates, and isotropic dis­place­ment values.

The calculated 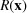 values are plotted in two different views: a top view and a three-dimensional fly-by. Also, the lowest 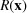 value of each plane is marked with a brown sphere.

This lowest minimum of a cut will be called ‘cut lowest minimum’ (CLM) from here on, while the minimum with the lowest possible *R* value will be referred to as the ‘global minimum’ (GM). This is 0.0 for a perfect match, meaning that all scatterers are in the correct position with respect to the model structure.

Additionally one can apply a resolution filter^3^ to the set of individual reflections *hkl* and remove all of those with lattice plane spacing 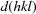 (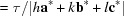, with the reciprocal lattice vectors 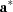, 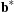, 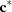) smaller than a chosen resolution cutoff 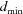. So, only reflections with 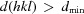 are used in the calculation of 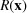.

*Xtal-xplore-R* is also intended as a didactic tool to help students visualize how changes in atom parameters affect the residual function and can be used to demonstrate manual structure determination for some simple crystal structures.

### 3.1. Implementation   

Owing to its widespread use, good availability, multi-platform support and open-source code we chose Python (van Rossum, 2010 ▸) and PyQT4 (Riverbank, 2014 ▸) to implement the graphical user interface (GUI) of *Xtal-xplore-R* using *Qt Designer* (http://www.qt.io/). For three-dimensional visualization we use *Mayavi 2* (Ramachandran & Varoquaux, 2011 ▸), a powerful *VTK* (Kitware, 2014 ▸) based cross-platform visualization toolkit. Quite a lot of the crystallographic functionality has been implemented using *cctbx* (Grosse-Kunstleve *et al.*, 2002 ▸, 2014 ▸). This currently forces our program to use Python 2(.7) despite our effort to keep Python 3 compatibility in mind.

### 3.2. Overview of the interface of *Xtal-xplore-R*   

**Figure 1:**
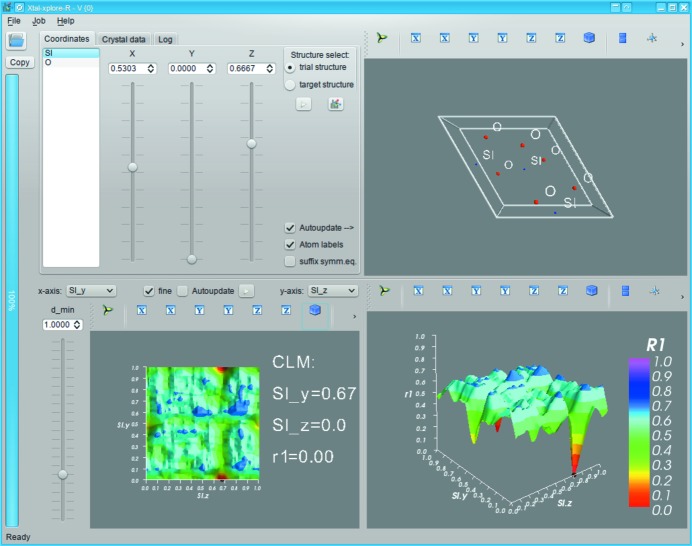
Screenshot of *Xtal-xplore-R*.

*Xtal-xplore-R* uses a single-window interface as depicted in Fig. 1 ▸ for its GUI to display all the relevant information at one time simultaneously. As *Xtal-xplore-R* is still under active development it is likely that some functions will be added or renamed in between the writing of this paper and its publication. Also, future versions may have additional features or a refined GUI. At the time of writing, the main elements of the GUI are as follows:

#### 3.2.1. The outer elements   

The outer elements are a menu bar to select different (advanced) program functions at the very top and buttons for general functions (Open CIF, Copy currently selected structure to the other structure slot) and a progress meter to display the progress of actions that take some time to complete to the very left. At the very bottom of the window there is a status bar to display some additional information to the user.

#### 3.2.2. The inner part   

The inner part of the GUI is divided into four sections. The top left is used for displaying information on and acting upon the two crystal structures, while the top-right three-dimensional visualization displays a structural model of the currently selected crystal structure. The two bottom visualization widgets are used to render surface plots of the crystallographic residual function in the selected cut plane. The *x* axis and *y* axis selectors on top of the bottom-left widget can be used to select which two-dimensional cut of the *m*-dimensional hyperspace should be displayed. The ‘fine’ toggle changes the resolution of the grid on which 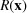 is evaluated from 0.1 steps to 0.01 steps, while the ‘
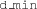
’ slider can be used to set the resolution cutoff.

#### 3.2.3. Basic operation   

Usage instructions and a detailed example workflow can be found in the manual that is supplied along with *Xtal-xplore-R*.

In a nutshell, *Xtal-xplore-R* allows the user to load up to two CIFs and then manipulate the different atomic coordinates at will while displaying the resulting ‘landscapes’ of the selected cut through the residual function. (In most cases one will only load one CIF and use it as target structure and as a base for the trial structure.) The interface is designed to be intuitively usable with some basic knowledge of crystal structure plotting.

### 3.3. Obtaining *Xtal-xplore-R*   

The source code, installation instructions and all documentation of *Xtal-xplore-R* can be obtained from https://github.com/jamasi/Xtal-xplore-R. The interested reader is kindly asked to clone this repository and to submit enhancements or bug-fixes as pull requests.

## 4. Simple example structures   

To demonstrate the use of *Xtal-xplore-R* we discuss briefly the target functions of three simple structures: high quartz (SiO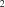) (Kihara, 1990 ▸), perovskite (CaTiO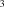) (Beran *et al.*, 1996 ▸) and wuestite (FeO) (Fjellvag *et al.*, 2002 ▸).

The structures were chosen as they (*a*) are widely known examples of inorganic crystals, (*b*) nicely show the characteristic properties of the target function and (*c*) contain only a few independent atoms, as this helps to keep the calculation times short. The quite high symmetries also help in checking if effects introduced by pseudo-symmetry are visible after the structures have been expanded into *P*1.

### 4.1. Some two-dimensional cuts in three-dimensional plots   

**Figure 2:**
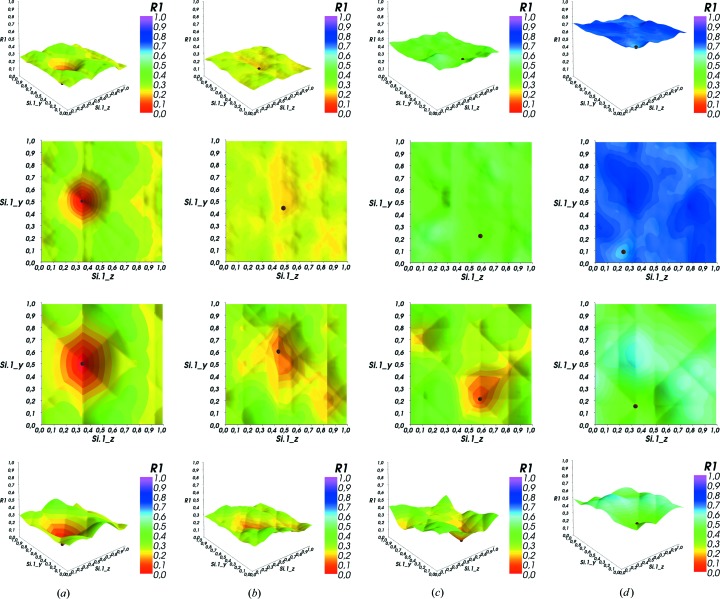
Effect of the proximity to the correct solution for high quartz (SiO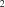) in *P*1 at two different data resolutions: (top) 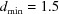
 Å, (bottom) 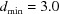
 Å. The brown sphere marks the position of the CLM. (*a*)–(*d*) are described in the text.

The plots in Fig. 2 ▸ show sections though the 27-dimensional hyperspace of the residual function of high-quartz-derived structures (in *P*1) for the *y* and *z* parameters of the Si.1 ion (the scatterers before expansion to the *P*1 symmetry equivalent are labelled with ‘Sc.*x*’ notation). The small brown sphere marks the CLM.^4^

The cuts were made for different target structures:

(*a*) Containing the solution: all atoms are placed on exact positions, so only the two parameters shown below differ from the optimal configuration.

(*b*) Very close to the solution: only Si.1 is placed on a wrong position (0.8 0.8 0.13); all other atom positions are correct but rounded to 0.01.

(*c*) Close to the solution: only Si.2, O.0 and O.3 are placed on random positions; all other atom positions are correct but rounded to whole tenths to add some more ‘noise’.

(*d*) Far away from the solution: all atoms of the target structure except Si.0 (0.5 0.0 0.0) are placed on random positions (= fractional coordinates 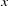, 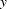, 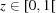).

Also, all plots were generated for two different values for the data resolution filter. The top row shows the *R*-factor landscapes for 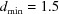
 Å, while the bottom row uses a lower data resolution with 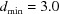
 Å.

**Figure 3:**
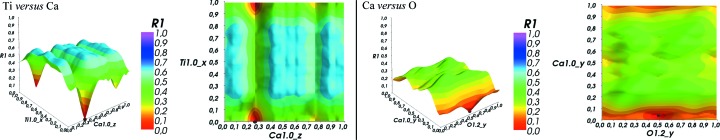
CaTiO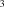 (perovskite). The effect of the heavy Ti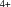 ion compared to the lighter Ca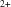 ion and the even lighter O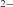 ion. The heavier the scatterer, the deeper the trench.

The second group of plots (Fig. 3 ▸) shows two different slices through the target function of a CaTiO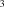 perovskite structure. One can clearly observe trenches with the deepest of those containing the GM in their intersection. In addition, the effect of the different ‘weight’ of the scatterers can be seen: the heavier the scatterer, the more pronounced and deeper the trench that denotes the match of one of its atomic positions. This can also be seen in many more plots from the complete data set.

**Figure 4:**
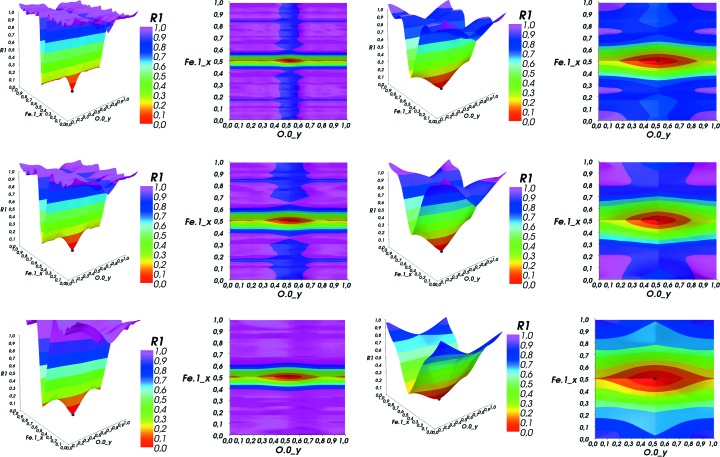
Effect of successive 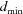 filtering on the Fe1
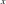
–O0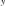 cut plane of wustite. The minimum gets wider while the whole surface gets successively smoother. Also notice the much deeper trench of the Fe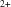 ion.

In Fig. 4 ▸ the same parameter plane from a wuestite (FeO) sample is plotted for different values of the minimal *d*-space resolution (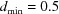, 0.8, 1.0, 1.5, 1.8 and 2.0 Å). As one can see, the global minimum does not change its position while getting successively wider and the surface of the cut gradually flattens.

### 4.2. Observations and their consequences   

Even from a brief look at the generated 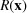 ‘landscapes’ of these example structures a number of the postulates on the properties of the target function made above can be verified:

(*A*) It is multidimensional. This is trivial.

(*B*) It is multi-modal. On most cuts there is more than one (local) minimum. Indeed, the residual function is multi-modal in any parameter direction except, usually, for the scale factor (not shown).

(*C*) It is band limited. Filtering the reflection list to structure factors above a cutoff in *d* spacing (= lower resolution in 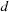 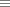 higher cutoff 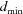) by removing all reflections *hkl* with too small 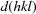 from the *R*-factor calculation flattens the landscapes and widens the minima without shifting their position in parameter space. Owing to the widening of the minima, the number of smaller local minima decreases as neighbouring minima merge into each other.

Such a filtering should help to reach global convergence faster, as this filtering process can be controlled dynamically from an algorithm.^5^

The fact that the underlying Fourier series is band limited has the following implications: The data are necessarily incomplete. This is a disadvantage if the refinement of very precise coordinates is required. On the other hand, the ‘band limitedness’ imposes an upper bound on the slope of the target function. Indeed, for the purpose of global optimization, where the primary goal is to generate an approximate solution which is close enough to the suspected optimum to subsequently let local optimizers succeed, ‘band limitedness’ can be turned into an advantage: The resolution can be reduced arbitrarily below the resolution of the experimental data by cutting off high-order Fourier coefficients from the data. This obviously reduces the accuracy of the optimized parameters and may cause, for severe band limitation, a loss of uniqueness of the solution, but it also increases the convergence volume in parameter space (corresponding to the number of extrema per unit interval) without changing the position of the global optimum. Adjustment of the data cutoff is therefore always a trade-off between the computational effort necessary to come close to the solution and the accuracy/uniqueness of the solution obtained.

(*D*) The variables are not separable. Moving away from the GM (in the parameters that are not displayed) successively increases the base level of the cut (see Fig. 2 ▸). Also, the position of the lowest minimum in the cut (CLM) no longer corresponds to the GM for large parameter deviations. This clearly shows that the parameters of this type of optimization problem are not separable. (Separability in this context would mean that any cut along any parameter direction should have its local optimum at the position of the global minimum for this parameter, irrespective of the values of any other parameters.) The position of the CLM locks in gradually and converges to the GM. In addition, trenches along the parameter directions or the diagonals gradually form, with the deepest (*i.e.* most pronounced) trench in each direction containing the solution. The ‘heavy’ atoms form deeper trenches compared to ‘lighter’ atoms. This can be interpreted as a kind of ‘semi-separability’ close to the GM.

(*E*) The residual function is noisy owing to 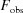 being quantities with a non-vanishing standard deviation. The result of different levels of ‘artificial noise’ is yet to be explored, but the quality of standard laboratory data is always sufficient (given suitable starting coordinates) to allow convergence of local optimizers to the global optimum. There is, therefore, no obvious reason why statistical data quality should be an issue for global optimization against the same data.

## 5. Outlook   

Further extensions of *Xtal-xplore-R* are expected to include the option to simulate powder diffraction, the ability to add user-defined noise on simulated data, the use of intensity data supplied by the user, and a fast local optimizer to explore the convergence radius of the global minimum.

The GUI development for *Xtal-xplore-R* along with its underlying crystallographic routines will also be used in the implementation of the optimization algorithms, taking advantage of the above-mentioned observations.

One of these is the successive line scan (SLS) algorithm that will try to locate trenches within low-dimensional cuts through the parameter space and then in combination with a local optimizer try to successively descend towards the GM. The above-mentioned advantage of controlling the resolution cutoff gives rise to an alternative algorithm: the optimal configuration search (OCS). In this algorithm, the atomic positions manifold (and therefore parameter space) is discretized into a set of only a few allowed positions. These positions can then be either occupied or not, thus transforming the continuous problem into a combinatoric one, where one can iterate all possible combinations in a significantly shorter time.

## Footnotes

1 Diffraction data are incomplete in various respects: (*a*) The underlying Fourier series is always finite (band limited) because only a limited quantity of data is experimentally accessible. (*b*) Only the structure factor amplitudes are measurable; the corresponding phases are missing (the ‘phase problem’ of crystallography). (*c*) For powder diffraction data, orientational averaging leads to systematic and/or occasional superposition of observations; only their sum is observable.

2 For  and a fast computing system which could check a million grid points per second it would take  s to evaluate the complete grid. This is more than twice as long as the age of the universe (about  s).

3 We call this filter a ‘resolution filter’ as it simulates the cutoff of observable reflections due to limited XRD instrument resolution.

4 Owing to space limitations on this paper only this single example picture is shown here, but the observations presented below are based on the full set of all parameter plots.

5 The isotropic displacement parameter has an influence that is quite similar to that of the data cutoff and is not discussed in this paper for brevity.
